# Drug Resistance and the Role of Combination Chemotherapy in Improving Patient Outcomes

**DOI:** 10.1155/2013/137414

**Published:** 2013-06-24

**Authors:** Denise A. Yardley

**Affiliations:** Breast Cancer Research, Sarah Cannon Research Institute, 250 25th Avenue North, Suite 110, Nashville, TN 37203, USA

## Abstract

Resistance to cancer chemotherapy is a common phenomenon especially in metastatic breast cancer (MBC), a setting in which patients typically have had exposure to multiple lines of prior therapy. The subsequent development of drug resistance can result in rapid disease progression during or shortly after completion of treatment. Moreover, cross-class multidrug resistance limits patient treatment choices, particularly in a setting where treatments options are few. One attempt to minimize the impact of drug resistance has been the concurrent use of two or more chemotherapy agents with unrelated mechanisms of action and differing modes of drug resistance, with the intent of blocking the development of multiple intracellular escape pathways essential for tumor survival. Within the past decade, an array of mechanistically diverse agents has augmented the list of combination regimens that may be both synergistic and efficacious in pretreated MBC. The aim of this paper is to review mechanisms of resistance to common chemotherapy agents and to consider current combination treatment options for heavily pretreated and/or drug-resistant patients with MBC.

## 1. Introduction

Metastatic breast cancer (MBC) is a heterogeneous disease and among the leading causes of cancer mortality, accounting for more than 400,000 deaths annually worldwide [1]. The aim of this article is to review mechanisms of drug resistance to common chemotherapy agents and current combination treatment options for heavily pretreated, drug-resistant, or refractory patients with MBC.

### 1.1. Prognostic and Predictive Value of Breast Cancer Subtypes

Patients with MBC have a poor prognosis, and optimal therapeutic regimens are yet to be established. However, in recent years, the introduction of new chemotherapeutic regimens has led to modest improvements in survival [2]. Current treatment algorithms take into account the tumor expression of human epidermal growth factor receptor 2 (HER2) and/or hormone cell surface receptors. Expression of these receptors as well as unique gene expression patterns, identified through genomic profiling studies, can be used to further categorize breast cancer into at least five common subtypes: luminal-like subtypes A and B (expression of hormone receptors and luminal cytokeratins 8 and 18), basal-like (expression of cytokeratins 5 and 17 and typically no expression of hormone or HER2 receptors), HER2-positive or -enriched (mostly, but not all, HER2 amplified), and normal-like [3–5]. 

 The patterns of gene expression are hoped to one day carve out individual treatment choices. For instance, triple-negative breast cancer (TNBC), a clinical phenotype characterized by the lack of estrogen receptor (ER), progesterone receptor, and HER2 tumor expression, carries an extremely poor prognosis and frequently demonstrates a basal-like or claudin-low genomic profile [6]. Its high proliferative index makes TNBC susceptible to chemotherapy although defects in DNA repair underlie the high rate of rapid relapse [7, 8]. For the hormone receptor-positive cancer, further division into a luminal A signature identifies an extremely favorable hormone-sensitive tumor best suited to an antiestrogen treatment strategy whereas luminal B tumors are characterized by a higher proliferative rate, higher rates of disease relapse and/or progression, and rapid development of endocrine resistance [9, 10]. Contributing to this resistance is ligand-independent growth factor signaling mediation by up-regulated epidermal growth factor, HER2, and/or insulin-like growth factor receptor 1 signaling pathways [11, 12]. HER2-positive MBC is sensitive to trastuzumab; however, despite initial responses, most tumors develop resistance within 1 year of treatment initiation and about 15% relapse after adjuvant trastuzumab [13]. A major determinant of these tumors' resistance is increased signaling via the phosphatidylinositol 3-kinase/Akt pathways [14]. Thus, tumor genomic signatures may determine the fate of a cancer cell after drug treatment, based on its capacity to develop drug resistance that may have occurred either before or after the tumorigenic transformation. Gene expression arrays may define the critical molecular and cellular mechanisms responsible for the drug-resistance phenotype.

Subclassification of newly diagnosed MBC into hormone receptor-positive cancer, HER2-positive MBC, or TNBC is well established as a means of assessing prognosis and treatment options [15, 16]. A more recent development is the use of multiparameter breast cancer gene-profiling assays, such as the Oncotype DX 21-gene assay, Breast Cancer Profiling assay, and MammaPrint test, to provide predictive and prognostic information [15, 17]. Two prospective clinical trials (Trial Assigning IndividuaLized Options for Treatment (Rx) (TAILORx); Microarray in Node-negative Disease may Avoid ChemoTherapy (MINDACT)) are underway to assess the utility of the Oncotype DX and MammaPrint tests in women with early stage disease [15, 17]. The potential value of gene-profiling assays in appraising response to therapy in patients with advanced breast cancer or MBC remains to be confirmed, and ultimately the clinical value of genomic predictors of response to therapy in MBC will depend on correlation of these signatures with clinical efficacy.

### 1.2. Treatment Selections in MBC

Generally, patients with hormone receptor-positive MBC and low tumor burden are prescribed antiestrogen endocrine therapy. However, in hormone-insensitive or refractory MBC, systemic cytotoxic chemotherapy is the standard approach [15]. Because very few widely accepted treatment algorithms exist for chemotherapy in MBC, selection of therapy is individualized and based on several factors including the tempo of the disease (i.e., whether there is rapid clinical deterioration, life-threatening visceral disease, overwhelming tumor burden, short disease-free interval, or the need for rapid symptom control); individual patient treatment goals, quality-of-life issues, and patient performance status.

 For decades, anthracyclines have been a standard treatment for women with advanced breast cancer; however, anthracycline administration is now largely confined to the adjuvant setting. In patients with MBC, anthracyclines are associated with only modest advantages in median time to progression (TTP) or progression-free survival (PFS), even in anthracycline-naïve patients [18–22]. Cumulative cardiotoxicity limiting lifetime exposure to anthracyclines, often preventing prolonged administrations and/or rechallenge in later lines of therapy [23], coupled with the increased pool of efficacious novel agents in MBC has led oncologists to consider alternative options in the first- and later-line MBC settings. First-line regimens for MBC now typically include taxanes, either alone or in combination, which demonstrate superior efficacy as compared with nontaxane anthracycline-based regimens [18, 20, 24]. Thus, nonanthracycline taxane-based regimens, with or without trastuzumab, have become a standard adjuvant therapy in both the HER2 normal and HER2+ early disease settings [15]. Now, as an accepted standard option in early stage disease, the pendulum may shift again regarding the use of anthracyclines in the metastatic disease setting. Indeed, several trials exploring novel therapies in MBC patients with unmet medical needs are mandating that these patients have demonstrated disease progression following exposures to both anthracyclines and taxanes in either early or later stage disease.

## 2. Drug Resistance

Studies have shown that more than one-third of patients with MBC do not respond to first-line anthracyclines or taxanes, and even with the addition of biologic agents, disease progression occurs within a median of less than 1 year [25–28]. One of the main clinical issues is the development of drug resistance, which accounts for failure of treatment leading to death in more than 90% of patients with MBC and affects all classes of agents (conventional cytotoxics, hormonal, targeted) [29]. For the purpose of guiding the selection of subsequent chemotherapy, the time to relapse after initial chemotherapy is empirically divided into 6-month blocks, with refractory tumors demonstrating progression during or immediately following chemotherapy. When the disease-free interval is brief (less than 6 months or even between 6 and 12 months), acquired or intrinsic drug resistance appears to be the culprit behind tumor progression [29, 30]. In general, the shorter the interval from chemotherapy to relapse, the less likely the subsequent courses will be effective long term. 

 As anthracyclines and/or taxanes are commonly used in the adjuvant breast cancer setting [15], the majority of patients with MBC are exposed to these agents by the time of their initial recurrence. Rechallenge with the same agents may be successful in some patients. The exact time period between the initial exposure and rechallenge is not clearly established; by convention, those relapsing after longer than 12 months are considered “sensitive,” and those relapsing within 6 to 12 months may have acquired resistance and thus benefit from a different class of agent [31]. Although response rates and duration of response progressively decline with subsequent lines of treatment, recent improvements in the survival of patients with MBC are likely attributable to the increasing number of lines of therapy deliverable and the greater availability of novel agents [32]. Optimizing the efficacy of subsequent therapy for patients with resistant MBC will undoubtedly depend on detailed analysis of the mechanism of resistance. 

### 2.1. Mechanisms of Drug Resistance

Resistance can manifest as a result of decreased drug activity and can be primary (present prior to drug exposure, with the tumor insensitive to initial treatment) or acquired (tumor resistance developing during or after the course of treatment). This innate and/or adaptive resistance to chemotherapy critically limits treatment outcomes and remains a key challenge for clinicians. Because a particular mechanism can affect agents from completely unrelated classes of chemotherapy agents, subsequent treatment options may be compromised [33]. This so-called multidrug-resistance phenomenon constrains the efficacy of anthracyclines, taxanes, and several other cytotoxic and biologic agents [34]. On a cellular level, there are multiple active mechanisms of resistance, which include increased activity of efflux pumps, such as adenosine-triphosphate- (ATP-) dependent transporters or reduced drug influx; activation of detoxifying proteins, such as cytochrome P450 mixed-function oxidases; activation of mechanisms that repair drug-induced DNA damage; and disruptions in apoptotic signaling pathways, which may decrease susceptibility to drug-induced cell death [33].

Alterations in drug efflux mechanisms are a common cause of multidrug resistance [34]. Overexpression of ATP-binding cassette (ABC) transporter proteins, such as P-glycoprotein (P-gp), multidrug-resistance-associated protein 1 (MRP1), and breast cancer resistance protein, can all be involved [35]. ABC transporters act by pumping anticancer agents out of the intracellular milieu into the extracellular matrix, thereby preventing the agents from reaching their minimally effective intracellular concentrations [34]. Substrates for P-gp include anthracyclines, taxanes, antimetabolites, and vinca alkaloids [35]. In a meta-analysis of 31 breast cancer clinical trials, overexpression of P-gp was associated with 3-fold increased risk of failure to respond to chemotherapy and its expression was noted to increase after chemotherapy exposure [36]. Although MRP1 expression is high in chemotherapy-naïve tumors, its expression has also been shown to increase with exposure to cytotoxic agents [35]. MRP1 has been shown to confer resistance to anthracyclines and vinca alkaloids, but not taxanes [35].

 Major determinants of taxane efficacy are changes in ABC transporter activity along with alterations in *β*-tubulin, such as shifts in tubulin isoform expression patterns and development of mutations [34]. Overexpression of the *β*III-tubulin isoform negatively correlates with taxane response because of its ineffective *β*III-tubulin binding [37]. In fact, high expression of *β*III-tubulin correlates with cancer progression on paclitaxel treatment [38]. Furthermore, recent evidence reveals that *β*III-tubulin overexpression may be more common in certain breast cancer subtypes, including HER2-positive and basal-like/triple-negative, and may contribute to their aggressiveness [39].

*Tau*, one of the most extensively studied microtubule-associated proteins, binds to and stabilizes microtubules [40]. *Tau* controls microtubule stability through isoforms and phosphorylation. In vitro, low expression of *tau* rendered microtubules more vulnerable to taxane treatment, whereas microtubules assembled in the presence of *tau* were less susceptible to drug binding [41]. In a recent MBC study, *tau* expression was associated with a shorter TTP than *tau* negativity (6.0 versus 9.4 months, resp.) [42]. Likewise, in a phase II neoadjuvant study, *tau* gene expression inversely correlated with sensitivity to ixabepilone [43]. Although the mechanisms of action are similar between the taxanes and the epothilones, clinical data support at least a partial noncross resistance between the classes [44], and biomarkers might contribute to the identification of benefit from one class of microtubule-targeting agents or even a single agent. Large-scale pharmacogenomic analyses are underway to identify additional molecular markers potentially capable of distinguishing tumors with differential sensitivities to taxanes and ixabepilone. A recent study was successful in deriving genomic predictors for taxane and ixabepilone sensitivity from breast tumor cell lines [45].

 The chemotherapy-resistant phenotype of cancer cells is by no means the result of a single gene aberration. Although multidrug and taxane resistance mechanisms are among the best characterized, agents targeted against multidrug resistance have yielded poor results. Since it is recognized that multiple genetic defects—namely, DNA mutations, translocations, truncations, deletions, or duplications—are present in cancer cells, the role of drug-induced epigenetic aberrations such as histone posttranslational modifications, DNA hypermethylation, and subsequent gene silencing is of increasing importance [46–49]. Epigenetic changes can occur rapidly upon chemotherapy exposure. 

 Intrinsic or acquired resistance to biologic agents, such as trastuzumab [50], bevacizumab [51], and hormonal therapies [52], also occur not only in the specific pathways that underlie their respective mechanisms of action, but also through redundancy and cross-talk with other pathways that ultimately contribute to treatment failure [53, 54]. Parallel signaling through epidermal growth factor receptor (EGFR/HER1) is a factor in trastuzumab resistance, and cross-talk between the HER2 and ER*α* pathways can mediate resistance to antiestrogen therapies such as tamoxifen or the aromatase inhibitors [13, 55]. The dual kinase inhibitor lapatinib has already demonstrated the ability to overcome trastuzumab and tamoxifen resistance by virtue of its ability to block signaling through both HER2 and EGFR1, thus effectively reducing cross-talk between HER2 and ER*α* [53, 54]. 

 Changes that interfere with drug-target binding may also convey resistance to biologic agents. For example, trastuzumab resistance is affected by overexpression of MUC4, which hinders the antibody from binding to HER2 [13], or by expression of a highly active truncated shed form of HER2 (p95^HER2^) that lacks the extracellular trastuzumab-binding domain [53, 56]. Other factors that may play a role in resistance to targeted agents include decreased expression of the target inhibitors (e.g., PAX2), increased expression of exogenous ligands (e.g., transforming growth factor alpha), or decreased expression of inhibitory phosphatase (e.g., PTEN). Independent of these factors, downstream cellular changes such as increased or decreased expression or mutation of key signaling cascade components or downstream effectors (e.g., Ras/PI3K/PTEN/Akt/mTOR, Ras/Raf/MEK/ERK, and p53 pathways) can also determine the outcome of chemotherapy or targeted therapy [13, 57].

### 2.2. Strategies to Overcome Drug Resistance

Mechanisms of intrinsic drug resistance and the acquisition of resistance with disease progression are profoundly influenced by the tumorigenic pathway. Since both genetic and epigenetic changes are not static, they act in concert to maintain the malignant “homeostasis.” Many attempts to circumvent mechanisms of resistance to cytotoxic, hormonal, and biologic agents have failed. Approaches that have evaluated concomitant administration of agents that divert resistance have demonstrated limited clinical efficacy [58, 59]. More recently, the incorporation of mTOR inhibitors, histone deacetylase inhibitors, or demethylation agents has had some success in preclinical and clinical studies [14, 60–63].

Although tumors relapsing on anthracycline- or taxane-based adjuvant therapy regimens may be suitable for rechallenge [31], patients with resistant tumors more commonly receive an additional chemotherapy agent that demonstrate somewhat differing mechanisms of action and/or biochemical structures. These combinations may also demonstrate synergism not only in efficacy parameters but also in delaying disease progression by impacting on multiple intracellular escape pathways essential for tumor cell growth and survival. In recent years, an ever-growing array of mechanistically diverse cytotoxic agents with efficacy in MBC has been evaluated in combination treatment strategies.

## 3. Combination Chemotherapy Regimens

The genetic alterations that enable a cancer cell to acquire resistance provide the basis for clinical studies of novel drugs with a focus on “antiresistance” combinations. Virtually all curative chemotherapy regimens employ multiple agents. The majority of current combination regimens have been developed empirically and although patterns of cross-resistance, overlapping drug toxicity, and mechanisms of action are considered when designing such regimens, formal preclinical testing has played only a minor role. A mathematical model has been devised to describe the potency of combining chemotherapy and immunotherapy; however, it remains undefined whether most combinations are effective due to additive or synergistic cytotoxicity [64].

 The most “classic” drug combinations explored to date in the clinical disease settings are only additive or, at most, minimally synergistic. The traditional approach for the introduction of new agents has been to add the drug to accepted and/or established regimens. Since the leading goal of combination therapy is to attain therapeutic synergism, the search continues for the ideal drug partners that will act in this way to maximize tumor responses while offsetting tumor progression.

 Since sequential single agents in patients with advanced breast cancer is a common treatment strategy, the lack of evident increased response rates or improved PFS provides a strong rationale for continuing to explore combination regimens. A retrospective analysis of 1581 women with MBC who received first-line chemotherapy (doxorubicin plus an alkylating agent) found that, although complete responses (CRs) were uncommon and short lived (8–14 months), women who were treated with combination chemotherapy and achieved CRs were more likely to experience long-term survival [65]. Phase III trials that have shown that combination therapy also improves clinical outcomes in patients with MBC who have prior exposure to anthracyclines and/or taxanes are summarized in Table 1 [66–73] and Table 2 [25, 26, 74–79]. As noted, however, the majority of clinical benefit with combination chemotherapy is reflected in response rates, with limited survival advantages and questionable quality-of-life benefits in many cases, particularly when factoring in crossover or sequential exposure to the single agent [80]. The European School of Oncology Metastatic Breast Cancer (ESO-MBC) Task Force reported a preference for sequential cytotoxic monotherapy over cytotoxic doublets in advanced disease, with the caveat that patient- and disease-related factors must be taken into account when choosing between these two regimens [81]. Specifically, the risk of increased toxicity may be outweighed by the immediate benefit of higher response rates in the presence of rapid clinical progression, life-threatening visceral metastases, or the need for rapid symptom and/or disease control [81].

## 4. Taxanes Plus Third-Generation Cytotoxic Combination Regimens

### 4.1. Taxanes Plus Capecitabine

Because capecitabine has a low potential for myelosuppression, it is regarded as a preferred chemotherapy partner for several classes of cytotoxic agents. A preclinical rationale for combination with taxanes also exists as these agents have been shown to upregulate the tumoral activity of thymidine phosphorylase, an enzyme involved in the activation cascade of capecitabine; coadministration of capecitabine and either docetaxel or paclitaxel in preclinical models also demonstrated synergistic antitumor activity [82]. Based on its efficacy (overall response rate (ORR), PFS, and overall survival (OS)) and manageable adverse event profile in a phase III study, the doublet of capecitabine and docetaxel was approved for use in patients with MBC in whom prior anthracycline-containing chemotherapy had failed [66, 67]. The combination of capecitabine and paclitaxel has also shown activity in phase II trials in this setting [68, 69] (Table 1). 

In addition, capecitabine has been studied in combination with newer taxane formulations including nanoparticle albumin-bound (nab)-paclitaxel, which may have greater tumor penetration with improved efficacy and tolerability over standard paclitaxel. A combination of nab-paclitaxel and capecitabine (administered every 3 weeks) was evaluated in an open-label, phase II study of patients with MBC who were treated in first-line. ORR was 47.5%; CR occurred in 8% of 38 evaluable patients and stable disease in 39.4% [83]. Principal grade 3-4 toxicities were fatigue, hand-foot syndrome, gastrointestinal adverse events, and neutropenia, including febrile neutropenia.

### 4.2. Taxanes Plus Gemcitabine

Like capecitabine, the partially nonoverlapping tolerability profile and different mechanism of action of gemcitabine make it suitable for combination therapy with taxanes [84]. 

Combination therapy with gemcitabine plus paclitaxel is approved for first-line treatment of MBC in patients who have progressed after an anthracycline-containing adjuvant regimen. This approval is based on phase III results of first-line treatment with gemcitabine plus paclitaxel in patients with MBC exposed to adjuvant anthracyclines. The addition of gemcitabine to paclitaxel significantly extended median patient survival over single-agent paclitaxel (18.6 versus 15.8 months, *P* = 0.0489; adjusted Cox hazard ratio (HR): 0.78, 95% CI: 0.64–0.96, and *P* = 0.0187) [70] (Table 1) while making only minor contributions to the paclitaxel grade 3-4 toxicity profile. 

Noncomparative phase II studies in pretreated patients showed that gemcitabine plus docetaxel produced disease control rates of 73% to 74%, with TTP of 7 to 8 months and OS of 12 to 16 months [85, 86]. No phase III studies have evaluated the addition of gemcitabine to docetaxel compared with docetaxel alone in pretreated MBC patients. However, in a recent phase III study of anthracycline-pretreated patients, efficacy to gemcitabine plus docetaxel was equivalent to capecitabine plus docetaxel but was better tolerated [48] (Table 1).

 Similar to capecitabine, gemcitabine has been studied in combination with nab-paclitaxel. In an open-label phase II trial of patients with previously untreated MBC, weekly nab-paclitaxel plus gemcitabine was well tolerated and had significant activity [87]; ORR was 50%, median PFS was 7.9 months (95% CI: 5.4–10 months), and median OS was not reached.

## 5. Third-Generation Cytotoxic Combinations

### 5.1. Ixabepilone-Containing Combinations

Ixabepilone has antitumor activity in cells that have developed resistance to taxanes [88–90] and has a wider binding repertoire for the various isotypes of *β*-tubulin than the taxanes, including the taxane-refractory *β*III-tubulin isoform [88]. Moreover, the in vitro antitumor activity of ixabepilone is not affected by overexpression of multidrug-resistance proteins such as P-gp [89] or tubulin expression or mutations [44, 91], both of which may occur in response to paclitaxel treatment and both of which have been shown to confer resistance to paclitaxel. Synergistic antitumor activity has been demonstrated preclinically with ixabepilone in combination with capecitabine and other chemotherapies, including irinotecan and epirubicin [92].

Ixabepilone is currently approved for use in the United States as monotherapy for the treatment of locally advanced breast cancer or MBC after the failure of an anthracycline, a taxane, and capecitabine and in combination with capecitabine for the treatment of anthracycline- and taxane-resistant locally advanced breast cancer or MBC. Ixabepilone was approved in combination with capecitabine based on PFS and ORRs that were significantly higher than single-agent capecitabine (Table 1) in a large phase III clinical trial in patients with locally advanced breast cancer or MBC who were pretreated or resistant to anthracyclines and resistant to taxanes [72]. A confirmatory phase III study validated the results of this study with a similar patient population and design. In this trial, PFS was also prolonged with combination therapy (HR: 0.79; 95% CI: 0.69–0.90; *P* = 0.0005) [93]. However, OS data from both studies did not show statistical differences between the arms. In both studies, grade 3-4 treatment-related sensory neuropathy, fatigue, and neutropenia were more common in the combination therapy arm. 

Ixabepilone has or is being investigated in combination with several biologic agents. Together with carboplatin and trastuzumab in HER2-positive patients, ixabepilone administered first-line produced an ORR of 44%, TTP of 8.2 months, and median OS of 34.7 months [94]. In combination with bevacizumab, ixabepilone administered as weekly or every-3-week therapy in the first-line MBC setting demonstrated safety that was similar to that for weekly paclitaxel but with a higher response rate at 71% [95].

### 5.2. Vinorelbine-Containing Combinations

A number of combinations containing vinorelbine have been utilized in previously treated MBC, although none will be approved by the United States Food and Drug Administration (FDA) since vinorelbine is now available as a generic. One of the more common vinorelbine-based combination regimens is with gemcitabine, utilized based on data from a phase III trial of gemcitabine plus vinorelbine versus vinorelbine alone. The doublet significantly improved ORR and PFS over vinorelbine monotherapy, although this did not translate to longer OS [73] (Table 1). The toxicity profiles of gemcitabine and vinorelbine did not appear to overlap, but more grade 3-4 hematological toxicities were reported in the combination arm.

The combination of vinorelbine and trastuzumab is commonly used in MBC. A recent phase III study (HERNATA (HERceptin plus NAvelbine or TAxotere)) suggests that vinorelbine plus trastuzumab has comparable efficacy to docetaxel plus trastuzumab in terms of TTP, OS, and ORR, but with significantly fewer adverse events when used first-line in women with MBC [96]. 

### 5.3. Gemcitabine-Containing Combinations

Along with the previously mentioned combinations of gemcitabine plus vinorelbine or taxanes, the doublet of gemcitabine and carboplatin is increasingly being evaluated given the extensive use of taxanes in earlier stage disease. This combination has demonstrated significant preclinical synergy [97] and clinical efficacy in patients with HER2-negative, anthracycline- and taxane-pretreated MBC (ORR = 32%; TTP = 4.4 months; OS = 11 months) [98]. This combination has also been evaluated with or without trastuzumab (dependent on HER2 status) as first-line therapy for patients with MBC [99]. The ORR was 34% for patients with HER2-negative disease treated with gemcitabine/carboplatin and 66% for patients with HER2-positive disease treated with gemcitabine/carboplatin/trastuzumab [99]. 

A recent phase II study evaluated the efficacy of the poly(ADP-ribose) polymerase 1 inhibitor, BSI-201, in combination with gemcitabine/carboplatin in patients with metastatic TNBC [100]. The addition of BSI-201 to gemcitabine/carboplatin significantly improved the clinical benefit rate (34% versus 56%; *P* = 0.01) and ORR (32% versus 52%; *P* = 0.02), and prolonged median PFS (3.6 versus 5.9 months; HR: 0.59, *P* = 0.01) and median OS (7.7 versus 12.3 months; HR: 0.57, *P* = 0.01). However, the phase III data presented at the 2011 Annual Meeting of the American Society of Clinical Oncology (ASCO) showed that adding BSI-201 to gemcitabine/carboplatin in patients with metastatic TNBC failed to meet the prespecified criteria for significance for the coprimary endpoints of OS and PFS [101].

### 5.4. Eribulin-Containing Combinations

Eribulin mesylate, a synthetic analog of the marine sponge halichondrin B, is a nontaxane microtubule dynamics inhibitor with a distinct mode of action to that of other antitubulin agents [102, 103]. Potent preclinical activity in taxane-resistant tumor models [103, 104] resulted in clinical investigation of this agent [105–107]. Eribulin monotherapy was subsequently approved by the FDA in November, 2010 for the treatment of patients with MBC who have previously received at least two chemotherapeutic regimens for the treatment of metastatic disease; prior therapy should include an anthracycline and a taxane in either the adjuvant or metastatic setting. Approval was based on the phase III Eisai Metastatic Breast Cancer Study Assessing Physician's Choice Versus E7389 (EMBRACE) trial where significant improvement in median OS with eribulin was observed compared with treatment of physician's choice (13.1 months versus 10.6 months; HR: 0.81, *P* = 0.041) in patients with locally recurrent or MBC [108].

Eribulin is currently undergoing phase II investigation in combination with several other agents. These include trastuzumab as first-line treatment in patients with locally recurrent or metastatic HER2-positive breast cancer (NCT01269346), lapatinib in HER2-positive MBC pre-treated with trastuzumab (NCT01534455), and carboplatin as neoadjuvant therapy in patients with TNBC (NCT01372579).

## 6. Chemotherapy Plus Biologics or Targeted Therapy Combinations

Although this paper does not describe in detail the rationale for adding biologic therapy to chemotherapy, a brief discussion of biologics and their role in combination therapy, as well as an overview of biologics-only combinations, is provided. 

### 6.1. Trastuzumab-Containing Combinations

In patients with HER2-positive breast cancer, adding trastuzumab to anthracycline- or taxane-based chemotherapy in the adjuvant or first-line metastatic settings improved response rates and disease-free survival over chemotherapy alone [25]. In the adjuvant setting, its use has become standard following doxorubicin and cyclophosphamide when administered in combination with adjuvant taxane-based chemotherapy or when administered in combination with docetaxel/carboplatin, improving OS [109, 110].

 In two phase II studies, trastuzumab was active as first- or second-line MBC therapy when added to docetaxel plus either cisplatin (ORR = 79%; TTP = 9.9 months) or carboplatin (ORR = 58%; TTP = 12.7 months) [111]. Similar results were observed in a phase II trial combining trastuzumab with paclitaxel and carboplatin [112]. Subsequently, a phase III trial showed that addition of carboplatin to trastuzumab plus paclitaxel significantly improved ORR and median PFS but only in tumors that were clearly HER2-positive (immunohistochemical staining of 3+; ORR = 57% versus 36%; *P* = 0.03; PFS = 13.8 versus 7.6 months; *P* = 0.005) [113].

Despite contradictory preclinical reports, several clinical trials have demonstrated that the combination of capecitabine and trastuzumab is active and well tolerated in patients with anthracycline- or taxane-resistant, HER2-overexpressing breast cancer [114, 115]. Phase II trials have evaluated the addition of trastuzumab to several other cytotoxic therapies, including gemcitabine or gemcitabine/carboplatin [98, 116–118] and ixabepilone/carboplatin [94]. The results indicate these combinations are potentially active and well tolerated, but no comparative data are available. 

### 6.2. Lapatinib Plus Capecitabine

Lapatinib may be effective in overcoming some types of trastuzumab resistance. It is indicated with capecitabine for patients with HER2-overexpressing MBC following progression on anthracyclines, taxanes, and trastuzumab. In a phase III study, lapatinib plus capecitabine significantly prolonged TTP in its approved setting [75, 76]. An expanded indication for lapatinib in combination with letrozole has been also FDA approved for the treatment of hormone-positive and HER2-positive advanced breast cancer in postmenopausal women for whom hormonal therapy is indicated.

A combination of lapatinib plus nab-paclitaxel-administered first- or second-line to patients with HER2-positive MBC in a phase II trial produced ORR of 53% (95% CI: 41% to 66%), median PFS of 39.7 weeks (95% CI: 34.1 to 63.9 weeks), and an acceptable toxicity profile [119].

### 6.3. Bevacizumab Plus Capecitabine

The randomized phase III Regimens In Bevacizumab for Breast ONcology (RIBBON) 1 trial revealed that as first-line therapy, bevacizumab plus capecitabine outperformed single-agent capecitabine for response rate (35% versus 24%, *P* = 0.0097) and PFS (8.6 versus 5.7 months, *P* = 0.0002) [28]. Results from a recent meta-analysis of 3 randomized trials (E2100, AVADO (AVAstin-DOcetaxel) and RIBBON 1) in patients with MBC evaluating bevacizumab plus first-line chemotherapy regimens (taxane-, anthracycline-, or capecitabine-based) confirm the improvement in PFS with bevacizumab, but show no statistically significant difference in OS [120]. The results of a phase II study presented at ASCO 2011 suggested that bevacizumab plus capecitabine (with or without vinorelbine) is tolerable [121]. In the later setting, one randomized phase III study failed to demonstrate any survival advantage (PFS or OS) for patients with previously treated MBC receiving bevacizumab plus capecitabine compared with capecitabine monotherapy, despite significantly improved ORRs [74] (Table 2). In the phase III RIBBON 2 trial, however, the addition of bevacizumab to standard second-line chemotherapy (consisting of taxanes, gemcitabine, vinorelbine, or capecitabine as determined by investigators prior to randomization) significantly improved PFS (7.2 versus 5.1 months for chemotherapy alone; *P* = 0.0072) [122]. The benefits of the bevacizumab addition were consistent for all types of chemotherapy except vinorelbine, which was administered in only a very small group of patients. However, there was no statistically significant difference in OS between treatment arms [123]. Adverse events were consistent with previously reported observations [124].

The European Committee for Medicinal Products for Human Use recommended that the therapeutic indications of bevacizumab should be extended to include first-line treatment in combination with capecitabine for patients with MBC in whom treatment with other chemotherapy options, including taxanes or anthracyclines, is not considered appropriate. However, in December, 2010 the FDA recommended the removal of the breast cancer indication from the label for bevacizumab (in combination with paclitaxel first-line for HER2-negative MBC) because the drug has not been shown to be safe and effective. This decision was upheld in November, 2011 following an appeal from the manufacturer [125].

## 7. Combinations of Targeted and Biologic Therapies

Ongoing investigational trials continually aim to improve patient outcomes by combining chemotherapeutic and/or biological targeted agents for advanced MBC. A phase II trial evaluating trastuzumab plus bevacizumab reported a 48% response rate (24/50; two CRs) in HER2-positive patients undergoing first-line treatment for MBC [126]. The effect of biologic combinations in pretreated MBC is unclear, but interim results from phase II trials suggest that the combinations of lapatinib plus bevacizumab [127] and trastuzumab plus the mTOR inhibitor ridaforolimus [117] are both active and well tolerated in trastuzumab-pretreated HER2-overexpressing MBC. Three randomized, placebo-controlled, phase III trials are evaluating the mTOR inhibitor, everolimus, in the following combinations: with trastuzumab and paclitaxel as first-line therapy in HER2-positive MBC (Breast cancer trial of OraL EveROlimus (BOLERO) 1); with exemestane in postmenopausal women with ER-positive MBC who are refractory to letrozole or anastrozole (BOLERO 2), and with trastuzumab and vinorelbine in women with HER2-positive MBC who are resistant to trastuzumab and have been pretreated with a taxane (BOLERO 3). Recent results from the BOLERO 2 trial have shown that everolimus plus exemestane significantly improved PFS to 6.9 months compared with 2.8 months with exemestane alone (investigator assessment; *P* < 0.001) [62]. Furthermore, a recent randomized phase II study (TAMRAD (TAMoxifen-RAD001)) of everolimus plus tamoxifen showed that this combination provided significant improvement in the 6-month clinical benefit rate (61.1%, 95% CI: 46.9–74.1) compared with tamoxifen alone (42.1%, 95% CI: 29.1–55.9) in women with HR-positive, HER2-negative MBC with prior exposure to aromatase inhibitors [63].

One phase III trial has compared dual HER2 targeting therapy with trastuzumab plus lapatinib to lapatinib alone in heavily pretreated patients with MBC. This trial demonstrated increased PFS and OS in the combined arm, with both treatments being generally well tolerated [77, 78] (Table 2). In another trial of trastuzumab-pretreated patients with MBC, the combination of trastuzumab plus pertuzumab produced a 24% ORR (16/66; five CRs). Grade 3 toxicities resolved to the point where therapy could continue and there were no grade 4 adverse events [128]. The phase III CLinical Evaluation Of Pertuzumab And TRAstuzumab (CLEOPATRA) trial, which further investigated this regimen, met its primary endpoint with patients receiving pertuzumab and trastuzumab plus docetaxel experiencing significantly longer, independently assessed median PFS than those receiving only trastuzumab and docetaxel (18.5 versus 12.4 months, resp.; *P* < 0.001) [129]. Recent analysis of OS demonstrated a significant survival benefit in patients treated with pertuzumab and trastuzumab plus docetaxel [130].

## 8. Conclusion

Combination drug regimens with newer cytotoxic and biologic therapies are an effective strategy in fighting tumor growth and/or progression. These combinations can facilitate the attack on multiple intercellular processes, which may result in more efficient tumor responses. These strategies may also delay or circumvent mechanisms of drug resistance by interfering with cell survival or tumor growth pathways and the cross-talk established between them. Unfortunately, mechanisms of drug resistance to both cytotoxic and biologic therapies may ultimately limit the therapeutic efficacy of any anticancer drug, especially in heavily pretreated patients who have already exhausted many of their options. Although it is hoped that tumor gene expression profiles can help us select appropriate patients for specific treatments, development of drug resistance to chemotherapy or biologic therapy remains a major limitation. To this end, numerous ongoing and future trials are investigating novel effective combinations consisting not only of chemotherapy but also of biologic agents designed to target identified cell-signaling pathways that are active in settings of disease progression. Identifying additional biomarkers and potential drug targets may lead to the development of novel biologic/chemotherapy combinations that will ultimately extend the utility of these combinations. These advances in therapy will continue to help us overcome tumor resistance and disease progression in any given patient with breast cancer.

## Figures and Tables

**Table 1:** Combination cytotoxic therapy for patients with MBC pretreated with, or resistant to, taxanes or anthracyclines.

Therapeutic combination	Approval status	Mechanism of additive/synergistic action	Clinical data	Most common grade 3-4 adverse events
Docetaxel plus capecitabine (DX) [66, 67]	Phase III Approved	Taxane increases thymidine phosphorylase at tumor site, required for conversion of capecitabine to active 5-FU	*DX versus single-agent D* ORR: 42% versus 30% (*P* = 0.006) TTP: median 6.1 versus 4.2 months, HR = 0.652 (95% CI: 0.545–0.780; *P* = 0.0001) OS: median 14.5 versus 11.5 months, HR = 0.777 (95% CI: 0.645–0.942; *P* < 0.01)	*DX versus single-agent D* Neutropenic fever: 16% versus 21% Neutropenia: 16% versus 15% Stomatitis: 17% versus 5% Diarrhea: 14% versus 5% Nausea: 6% versus 2% Hand-foot syndrome: 24% versus 1% Alopecia: 6% versus 7% Fatigue/asthenia: 8% versus 11%

Paclitaxel plus capecitabine (PX) [68, 69]	Phase II Not approved	Taxane increases thymidine phosphorylase at tumor site, required for conversion of capecitabine to active 5-FU	*PX* [68, 69] ORR: 52%/81% TTP: median 8.1/8.4 months OS: median 16.5/21.6 months	*PX* [69] Hand-foot syndrome: 20% Neutropenia: 13% Fatigue: 7%

Paclitaxel plus gemcitabine (PG) [70]	Phase III Approved	Taxane arrests cell cycle for gemcitabine to induce cytotoxicity and stimulates apoptotic pathway	*PG versus single-agent P* RR: 41.4% versus 26.2% (*P* = 0.002) TTP: median 6.14 versus 3.98 months, HR = 0.70 (95% CI: 0.59–0.85; *P* = 0.002) OS: median 18.6 versus 15.8 months, HR = 0.82 (95% CI: 0.67–1.00; *P* = 0.019)	*PG versus single-agent P* Neutropenia: 47.5% versus 11.5% Anemia: 5.8% versus 1.5% Thrombocytopenia: 6.1% versus 0% Febrile neutropenia: 5.0% versus 1.2% Alopecia: 17.2% versus 22.0% Fatigue: 6.9% versus 1.2% ALT: 5.0% versus 0.4% Sensory neuropathy: 5.7% versus 3.9%

Docetaxel plus gemcitabine (DG) versus docetaxel plus capecitabine (DX) [71]	DG: phase III Not approved	Taxane arrests cell cycle for gemcitabine to induce cytotoxicity; taxane increases thymidine phosphorylase at tumor site, required for conversion of capecitabine to active 5-FU	*DG versus DX* RR: NSD PFS: NSD OS: NSD	*DG versus DX* Anemia: 6% versus 2% Neutropenia: 84% versus 79% Febrile neutropenia/neutropenic sepsis: 8% versus 4% Thrombocytopenia: 9% versus 4% Leukopenia: 77% versus 66% ALT/AST: 9% versus 5% Diarrhea: 8% versus 18% Nausea/vomiting: 10% versus 4% Mucositis: 4% versus 15% Asthenia: 7% versus 11% Hand-foot syndrome: 0% versus 26%

Capecitabine plus ixabepilone (XI) [72]	Phase III Approved	Synergism demonstrated preclinically, but a mechanism has not been defined	*XI versus single-agent X* ORR: 35% versus 14% (*P* < 0.0001) PFS: median 5.8 versus 4.2 months, HR = 0.75 (95% CI: 0.64–0.88; *P* = 0.0003) OS: median 12.9 versus 11.1 months, HR = 0.90 (95% CI: 0.70–1.05; NSD)	*XI versus single-agent X* Peripheral sensory neuropathy: 20.8% versus 0% Hand-foot syndrome: 18% versus 17% Diarrhea: 6% versus 8.5% Fatigue: 9% versus 3.3% Myalgia: 8% versus 0.3% Asthenia: 7.8% versus 0.8% Leukopenia: 57% versus 6% Anemia: 10% versus 4.5% Neutropenia: 68% versus 11% Thrombocytopenia: 8% versus 4%

Vinorelbine plus gemcitabine (VG) [73]	Phase III Not approved	Synergism demonstrated preclinically, but a mechanism has not been defined	*VG versus single-agent V* ORR: 36% versus 26% (NSD) PFS: HR = 0.66 (95% CI: 0.50–0.88) OS: HR = 1.04 (95% CI: 0.78–1.39; NSD)	*VG versus single-agent V* Neutropenia: 61% versus 44% Febrile neutropenia: 11% versus 6% Anemia: 6% versus 5% Thrombocytopenia: 8% versus 2% Alopecia (grade 2): 17% versus 17% Fatigue: 24% versus 17% ALT: 8% versus 3% AST: 5% versus 6% Prothrombin time or part prothrombin time alterations, or both: 7% versus 8% Constipation: 5% versus 2% Other infection: 9% versus 6% Dyspnea: 5% versus 6%

ALT: alanine aminotransferase; AST: aspartate aminotransferase; CI: confidence interval; FU: fluorouracil; HR: hazard ratio; NSD: not significantly different; ORR: overall response rate; OS: overall survival; PFS: progression-free survival; RR: response rate; TTP: time to progression.

**Table 2:** Randomized combination cytotoxic plus targeted or biologic therapy for patients with MBC.

Therapeutic combination	Approval status	Clinical data	Most common grade 3-4 adverse events
Trastuzumab plus chemotherapy (doxorubicin or epirubicin plus cyclophosphamide or paclitaxel) (TC) [25]	Phase III Approved	*TC versus C alone* ORR: 50% versus 32% (*P* < 0.001) TTP: median 7.4 versus 4.6 months (*P* < 0.001) TTF: median 6.9 versus 4.5 months (*P* < 0.001) DR: median 9.1 versus 6.1 months (*P* < 0.001) Death rate at 1 year: 22% versus 33% (*P* = 0.008) OS: median 25.1 versus 20.3 months (*P* = 0.046)	TC versus C alone* Leukopenia: 11% versus 9% Asthenia: 7% versus 7% Fever: 8% versus 4% Pain: 6% versus 7% Heart failure: 10% versus 2% Nausea: 5% versus 7% Vomiting: 5% versus 7% Alopecia: 26% versus 35%

Capecitabine plus bevacizumab (XB) [74]	Phase III Not approved in the United States; approved in the European Union	*XB versus single-agent X * ORR: 19.8 versus 9.1% (*P* = 0.001) PFS: median 4.86 versus 4.17 months, HR = 0.98 (*P* = 0.857) OS: median 15.1 months versus 14.5 months (NS)	*XB versus single-agent X* Diarrhea: 11.8% versus 10.7% Hand-foot syndrome: 27.5% versus 24.2% Hypertension: 17.9% versus 0.5% Thrombotic event: 5.6% versus 3.7%

Paclitaxel plus bevacizumab (PB) [26]	Phase III Not approved in the United States; approved in the European Union	*PB versus single-agent P* ORR: 36.9 versus 21.2% (*P* < 0.001) PFS: median 11.8 versus 5.9 months, HR = 0.60 (*P* < 0.001) OS: median 26.7 versus 25.2 months, HR = 0.88 (*P* = 0.02)	*PB versus single-agent P* Infection: 9.3% versus 2.9% Fatigue: 9.1% versus 4.9% Sensory neuropathy: 23.5% versus 17.7% Hypertension: 14.8% versus 0%

Capecitabine plus lapatinib (XL) [75, 76]	Phase III Approved	*XL versus single-agent X* ORR: 22% versus 14% (*P* = 0.09) TTP: median 8.4 versus 4.4 months, HR =0.57 (95% CI: 0.43–0.77; *P* < 0.001) OS: HR = 0.78 (95% CI: 0.55–1.12; *P* = 0.177)	*XL versus single-agent X [75]* Diarrhea: 14% versus 10% Hand-foot syndrome: 12% versus 14%

Lapatinib plus trastuzumab (LT) [77–79]	Phase III Not approved	*LT versus single-agent L* ORR: 10.3% versus 6.9% (*P* = 0.46) PFS: median 12.0 versus 8.4 weeks, HR = 0.77 (95% CI: 0.6–1.0; *P* = 0.029) OS: median 60.7 versus 41.4 weeks, HR = 0.74 (95% CI: 0.57–0.97; *P* = 0.026)	*LT versus single-agent L [79]* Diarrhea: 7% versus 7%

CI: confidence interval; DR: duration of response; HR: hazard ratio; NS: not significant; ORR: overall response rate; OS: overall survival; PFS: progression-free survival; TTF: time to treatment failure; TTP: time to progression.

*Percentage with severe event.
